# Psychometric performance of the Mental Health Implementation Science Tools (mhIST) across six low- and middle-income countries

**DOI:** 10.1186/s43058-022-00301-6

**Published:** 2022-05-19

**Authors:** Luke R. Aldridge, Christopher G. Kemp, Judith K. Bass, Kristen Danforth, Jeremy C. Kane, Syed U. Hamdani, Lisa A. Marsch, José M. Uribe-Restrepo, Amanda J. Nguyen, Paul A. Bolton, Laura K. Murray, Emily E. Haroz

**Affiliations:** 1 Johns Hopkins University Bloomberg School of Public Health, Baltimore, USA; 2 University of Washington Department of Global Health, Seattle, USA; 3 Columbia University Mailman School of Public Health, New York, USA; 4 University of Liverpool Institute of Population Health, Liverpool, UK; 5 Dartmouth Center for Technology & Behavioral Health, Lebanon, USA; 6 Pontificia Universidad Javeriana Department of Psychiatry and Mental Health, Bogota, Colombia; 7 University of Virginia School of Education and Human Development, Charlottesville, USA

**Keywords:** Mental health, Implementation measurement, Psychometrics, Low- and middle-income countries

## Abstract

**Background:**

Existing implementation measures developed in high-income countries may have limited appropriateness for use within low- and middle-income countries (LMIC). In response, researchers at Johns Hopkins University began developing the Mental Health Implementation Science Tools (mhIST) in 2013 to assess priority implementation determinants and outcomes across four key stakeholder groups—consumers, providers, organization leaders, and policy makers—with dedicated versions of scales for each group. These were field tested and refined in several contexts, and criterion validity was established in Ukraine. The Consumer and Provider mhIST have since grown in popularity in mental health research, outpacing psychometric evaluation. Our objective was to establish the cross-context psychometric properties of these versions and inform future revisions.

**Methods:**

We compiled secondary data from seven studies across six LMIC—Colombia, Myanmar, Pakistan, Thailand, Ukraine, and Zambia—to evaluate the psychometric performance of the Consumer and Provider mhIST. We used exploratory factor analysis to identify dimensionality, factor structure, and item loadings for each scale within each stakeholder version. We also used alignment analysis (i.e., multi-group confirmatory factor analysis) to estimate measurement invariance and differential item functioning of the Consumer scales across the six countries.

**Results:**

All but one scale within the Provider and Consumer versions had Cronbach’s alpha greater than 0.8. Exploratory factor analysis indicated most scales were multidimensional, with factors generally aligning with a priori subscales for the Provider version; the Consumer version has no predefined subscales. Alignment analysis of the Consumer mhIST indicated a range of measurement invariance for scales across settings (*R*^2^ 0.46 to 0.77). Several items were identified for potential revision due to participant nonresponse or low or cross- factor loadings. We found only one item, which asked consumers whether their intervention provider was available when needed, to have differential item functioning in both intercept and loading.

**Conclusion:**

We provide evidence that the Consumer and Provider versions of the mhIST are internally valid and reliable across diverse contexts and stakeholder groups for mental health research in LMIC. We recommend the instrument be revised based on these analyses and future research examine instrument utility by linking measurement to other outcomes of interest.

**Supplementary Information:**

The online version contains supplementary material available at 10.1186/s43058-022-00301-6.

Contributions to the literature

- Current implementation measures have limited appropriateness outside the high-resource, Western contexts where they were developed. We present a set of tools for the measurement of priority implementation outcomes and determinants with consumers and providers of mental health interventions in low- and middle-income countries.
- Use of these tools has rapidly outpaced psychometric evaluation. Our study expands the field of implementation science by being the first, to our knowledge, to examine the psychometric performance of implementation measures across multiple low- and middle-income countries.
- We provide evidence that these measures are reliable and internally valid and make recommendations for improvement and future research.

## Background

Substantial progress has been made over the past two decades to build an evidence base for mental health services within low- and middle-income countries (LMIC) [1]. Due to a dearth of specialist mental healthcare providers in many LMIC, much of the growing evidence base has focused on treatment approaches delivered by lay health workers. There are now dozens of randomized controlled trials (RCTs) supporting the effectiveness of psychotherapy delivered by nonspecialist health workers for depression, anxiety, posttraumatic stress, substance use, and violence [2–4], with over 20 RCTs focused on the implementation of the World Health Organization’s Mental Health Gap Action Programme alone [5]. Having established the effectiveness of interventions and approaches within research contexts, a major challenge facing researchers and practitioners is the implementation of this evidence base into mental healthcare systems of LMIC.

In mental health, as in many areas of health, the gap between evidence and practice is typically greatest in LMIC [6] and implementation science has been recognized as the next step in the evolution of global mental health [7, 8]. Implementation science aims to provide researchers and practitioners with tools to support the integration of evidence-based care into routine practice. Among these tools, quantitative implementation measures enable researchers to assess key determinants of implementation effectiveness and to evaluate implementation efforts according to the outcome domains defined by Proctor et al. of adoption, acceptability, feasibility, appropriateness, penetration, cost, fidelity, and sustainability [9, 10]. However, valid and reliable implementation measurement remains a challenge within mental health research in all contexts, due in part to the complexity of operationalizing concepts associated with implementation determinants and outcomes [11]. A review by Lewis et al. [12] identified 104 measures relevant to implementation science for mental or behavioral health. Of these, only one measure had minimal evidence for psychometric strength across six of their psychometric criteria, which include reliability, structural validity, criterion validity, norms, sensitivity to change, and length.

Mental health implementation research in LMIC is also impeded by the limited applicability of existing measures for use outside high-income contexts. Most implementation measures originate in Western high-income countries and rely on assumptions about healthcare that do not necessarily hold globally, often reducing their appropriateness for use within LMIC or lower-resource contexts within high-income countries [12, 13]. There are important differences in health system structures and financing, particularly in the role of insurance and payment mechanisms, between high-income countries and LMIC [14]. Mental health services in LMIC are frequently provided by nonspecialist health workers (e.g., nurses, community health workers, peers) rather than general physicians or specialist providers [15]. Evidence-based approaches to expanding mental healthcare coverage often rely on primary care or community-based platforms for service delivery; recipients of mental health interventions may rarely have contact with secondary or tertiary mental health facilities [16, 17]. Mental health implementation measurement is also limited because of choices in scale development; most implementation measures tend to be long and focus only on a single stakeholder group (e.g., intervention providers versus recipients), making their use less pragmatic for field research [11]. Because of these limitations, a major barrier to implementation science globally is the lack of pragmatic, accurate, and relevant implementation measurement, particularly valid and reliable implementation measures for global mental health.

In light of these challenges, researchers at Johns Hopkins University developed a series of measures to evaluate priority determinants and implementation outcomes for mental health interventions specifically in LMIC [18]. The Mental Health Implementation Science Tools (mhIST; pronounced “mist”) evaluate mental health interventions and programming according to the domains of implementation science defined by Proctor et al. [9] (Table 1). There are dedicated scale versions for each of three key stakeholder groups: (1) program consumers, (2) program providers, and (3) organizational level staff and leaders. The scale developers also adapted an additional measure for use in LMIC, the Implementation Leadership Scale [19], which was not evaluated in the current study.

**Table 1:** Mental Health Implementation Science Tools (mhIST) versions and scales

		Stakeholder version		
Scale domain	Simple description^b^	Consumer^a^	Provider^a^	Organization
Acceptability	Program is satisfying and agreeable for stakeholder	•	•	•
Adoption	Stakeholder is willing to try and then continue to use program	•	•	•
Appropriateness	Program fits your needs, culture, and values.	•	•	•
Feasibility	Program could be provided given available resources	•	•	•
Accessibility / Reach	Program would be easily available to people who need the services.	•	•	•
Organizational Climate	Environment in a workplace that affects employees well-being and performances	n/a	•	•
General Leadership	Program leadership supports, motivates, and enables people to achieve goals	n/a	•	•

^a^Included in current study

^b^Plan language description adapted from literature and presented to respondents

The mhIST is designed to be pragmatic and address limitations encountered when attempting to employ implementation measures developed for high-resource contexts. The original versions were pilot tested in Iraq and Myanmar [20, 21] which informed one round of revision (i.e., v1.0 to v2.0). After this initial process, Haroz et al. [18] validated the Consumer mhIST using a mixed-methods approach in Ukraine. Their qualitative findings informed further measure adaptation and led to the inclusion of additional context-specific items in Ukraine related to military veterans and their families. Their quantitative findings demonstrated good evidence for the internal reliability and criterion validity of the instrument using a vignette-based validation approach, where respondents used the mhIST to report on descriptions of high- and low-performing mental health programs. Internal consistency reliabilities of the instrument scales ranged from *α* 0.85 to 0.91 and test-rest reliabilities were acceptable to good for all scales (rho 0.61–0.79) [22, 23]. Total scale scores significantly differed by vignette assignment (odds ratios 2.21–5.6) and overall ratings (odds ratios 5.1–14.47), supporting criterion validity. While these initial validation studies have been limited, several researchers have begun employing the Provider and Consumer mhIST, including within research in South Africa, Kenya, Pakistan, and Sierra Leone [24–27]. In a recent study, Moore et al. used the tools to evaluate an opioid use prevention program in the USA because of the measures’ pragmatic characteristics and breadth of relevant implementation outcomes for community mental health [28].

Given the growing use of the mhIST, there is an urgent need to better understand its psychometric properties, particularly across diverse cultures and contexts. For this study, we evaluated the internal reliability, dimensionality, and individual item performance of the Consumer and Provider mhIST using secondary data from seven recent studies of mental health interventions in six LMIC: Colombia, Pakistan, Myanmar, Thailand, Ukraine, and Zambia. These study settings were selected based on data being complete and available at the time of analysis. Our objective was to establish the cross-context psychometric properties of these versions and inform future revisions. We then provide recommended revisions where individual items or scales did not perform acceptably.

## Methods

### Instrument development

Researchers from Johns Hopkins University populated the mhIST through four steps. First, they delineated and operationalized implementation determinants and outcomes in the context of LMIC, based on outcome domains defined by Proctor et al. [9, 10]: acceptability, adoption, appropriateness, feasibility, fidelity, reach, organizational climate, and leadership. Second, they mapped domains and constructs from two leading implementation science frameworks—the Consolidated Framework for Implementation Research [29]; and the Exploration, Preparation, Implementation and Sustainment framework [30]—to these outcomes. Third, scale developers solicited input on additional domains, constructs, content, and structure from experts in the field of international health, health systems, global mental health, and implementation science. Lastly, implementation domains and constructs were formulated into items with Likert scale response options.

The Consumer and Provider mhIST have been more commonly used in implementation research and are the focus of the present study (Tables S1 and S2). The Consumer version includes scales for the domains of Adoptability (AD; 9 items), Acceptability (AC; 17 items), Appropriateness (AP; 13 items), Feasibility (FS; 14 items), and Accessibility (RA; 8 items). The Provider version has a scale for each of these domains—Adoptability (9 items), Acceptability (13 items), Appropriateness (16 items), Feasibility (20 items), and Accessibility (9 items)—as well as scales for Organizational Climate (OC; 18 items), and General Leadership (GL; 9 items). Most domain scales of the Provider mhIST are further delineated into subscales, such as those distinguishing between the acceptability of the intervention and of individual professionalism within the Acceptability scale. Respondents are asked to rate each item using a four-point Likert scale with the options of “Not at all,” “A little bit,” “A moderate amount,” and “A lot.” Response options also include “Don’t know” and, when appropriate, “Not applicable.” Scales are then scored by calculating the response mean across all items for each scale. Researchers are also encouraged to review individual low-scoring items for potential program barriers and challenges, such as confidentiality concerns due to a lack of private space (item FS14).

#### Subjects and settings

We pooled data from seven studies to evaluate the performance of the mhIST within and across multiple LMIC (Table 2). Data included responses from consumers and providers of mental health interventions from most study sites. Only provider data are included from Lee et al. [21] because study authors made substantive changes to the Consumer mhIST given their target intervention recipients (i.e., consumers) were children. For the two studies in Zambia, researchers from one administered the mhIST only to providers [36] while in the other, researcher administered the mhIST only to intervention recipients [37].

**Table 2:** Context of studies included in the mhIST psychometric analysis

			Clients				Providers			
			Target population		Female	Age	Provider background		Female	Age
Study	Setting	Intervention		n	(%)	(Mean, SD)		n	(%)	(Mean, SD)
Marsch et al. [31]	Colombia	BA, PST, CBT	Adults with depression, AUD	117	76.9	54.7 (15.9)	Primary care providers	30	63.3	37.4 (7.8)
Lee et al., [20]	Karen State & Yangon, Myanmar; Mae Sot, Thailand	CETA	Adults affected by conflict or political persecution	198	78.3	36.0 (1.2)	Lay health workers	34	47.1	42.1 (2.2)
Lee et al. [21]	Kachin State, Myanmar	CETA	IDP youths	n/a	n/a	n/a	Lay health workers	18	77.8	27.6 (8.4)
Hamdani et al. [32]	Pakistan	Parents Skills Training	Caregivers of children with developmental delays	166	100	35.0 (7.6)	Caregivers of children with developmental delays	10	100	34.9 (4.4)
Murray et al. [33]	Ukraine	CETA	IDP and veterans	77	63.6	38.9 (9.9)	Psychologists, social workers, and lay health workers	30	86.7	41.0 (9.6)
Kane et al. [34]	Zambia	CETA	Families with IPV and unhealthy alcohol use	256	51.9	29.9 (11.2)	Lay health workers	n/a	n/a	n/a
Murray et al. [35]	Zambia	TF-CBT	Adolescents with HIV risk behaviors	n/a	n/a	n/a	Lay health workers	101	76.2	44.3 (11.2)

*AUD* Alcohol use disorder, *BA* Behavioral activation, *CBT* Cognitive behavioral therapy, *CETA* Common Elements Therapeutic Approach, *IDP* Internally displaced persons, *IPV* Interpersonal violence, *mhIST* Mental Health Implementation Science Tools, *n/a* not applicable (not included in study), *PST* Problem solving therapy, *SD* Standard deviation, *TF-CBT* Trauma-focused cognitive behavioral therapy

##### Myanmar and Thailand: adults

From 2011–2013, researchers from Johns Hopkins University conducted an RCT of the Common Elements Treatment Approach (CETA) among adult refugees and informal migrants from Myanmar in Mae Sot, Thailand [38]. CETA is a transdiagnostic adaptive treatment approach for mental and behavioral disorders developed to be delivery by lay counselors in settings with few mental health professionals [39]. Following the trial, Lee et al. [20] used the mhIST to study continued CETA implementation in Mae Sot as well as expand to Yangon and Karen State in Myanmar. A total of 198 participants across three sites completed the Consumer version, with all but one having fully completed the intervention at the time of response. Thirty-four lay mental health providers from the implementing organizations also completed the provider version. At the time of data collection, providers had completed an average of 48 CETA cases.

##### Myanmar: youths

Lee et al. also led a study of the adaptation and implementation of CETA for internally displaced youths in Kachin State, Myanmar [21]. Their research was motivated by concerns from stakeholders about youth mental health and requests for child-focused services from community-based organizations in northeastern Myanmar [40]. Lay health workers provided an adapted version of CETA to youths in six camps for displaced persons who had been exposed to conflict, violence, or other types of trauma and who met criteria for moderate to severe psychological distress. Eighteen providers from two implementing organizations who had been trained in CETA and received ongoing supervision for the duration of the study period completed the Provider mhIST. At the time of data collection, providers had completed an average of six CETA cases.

##### Ukraine

Murray et al. [33] conducted an RCT comparing brief and standard versions of CETA among those affected by conflict between pro-Russian separatists and Ukrainian loyalists stemming from the 2014 annexation of Crimea. Intervention recipients were adults who were internally displaced persons, military and para-military veterans, and others affected by conflict; all participants initially reported elevated symptoms of depression or posttraumatic stress and functional impairment. The mhIST were adapted through a qualitative study and previously validated in the study setting [18]. In the current study, 77 recipients completed the Consumer mhIST, with all but five having completed the intervention. Thirty providers who were Ukrainian psychologists, social workers, and lay health workers completed the Provider mhIST. At the time of data collection, providers had been delivering CETA for an average of 22.5 months and had an average of 11.1 years of experience working in mental health.

##### Colombia

Marsch et al. [31] conducted a modified stepped wedge implementation study of integrating mental health services into six primary care systems in Colombia. Their integration strategy relied on digital tools to detect, manage, and deliver services for depression and alcohol use disorder within primary care. Services provided during the study relied on elements of behavioral activation, problem solving therapy, and cognitive behavioral therapy delivered via digital platform, and included pharmacotherapy when indicated [31]. At the time of data collection, mhIST had been administered to 117 consumers at six and 12 months after being exposed to the mental health care model. Thirty nonspecialist primary care workers completed the Provider mhIST at the time they launched the mental health care model at their site and every six months thereafter for up to two years. We used available data from the most recent survey administration from each respondent in the present study.

##### Pakistan

Hamdani et al. [32] conducted an effectiveness implementation-hybrid randomized controlled trial of the World Health Organization Parents Skills Training program [41] in rural Pakistan. Caregivers of children with developmental delays received either skills training or enhanced treatment as usual, the latter including provider training in the detection and management of developmental disorders. One-hundred sixty-six caregivers completed the Consumer mhIST six months after program implementation. Providers were caregivers of children with developmental disorders who volunteered to be trained by trainers and provide skills training throughout the duration of the program; 10 providers completed the Provider mhIST six months after program implementation.

##### Zambia: families

Kane et al. [34] conducted an RCT in Zambia of CETA compared to treatment as usual plus safety checks among heterosexual families in which the woman reported recent interpersonal violence perpetrated by her current male partner and in which the male partner exhibited unhealthy alcohol use. The trial was ended early based on recommendation of the data and safety monitoring board due to an interim data analysis indicating a clear benefit of CETA at 12 months; participants in the control arm were then offered CETA [37]. Study investigators followed the original CETA participants for an additional 12 months for a 24-month post-baseline assessment [42]. Family members, including adolescents, in the CETA arm completed Consumer mhIST following their 12-month post-baseline assessment. Adolescent responses were included since no substantive changes were made to the instrument for different age groups. Providers were local lay counselors with no previous formal mental health training; they did not complete mhIST.

##### Zambia: youths

Murray et al. [35] completed an RCT of trauma-focused cognitive behavioral therapy compared to enhanced psychosocial counseling in reducing HIV risk behaviors among adolescents in Zambia. Intervention recipients were adolescents who were orphans or vulnerable children and who exhibited HIV risk behaviors. Providers were lay health workers who had at least a high school education and demonstrated basic communication and social skills; only one provider had previous training in mental health. Provider mhIST were administered to all 101 providers at the end of the study. Adolescent participants did not complete Consumer mhIST as a part of the study.

#### Analysis

##### Item comparison

We first reviewed translated versions of the instrument from each site to ensure site-specific cultural adaptations did not impede item cross-comparability. Researchers from five studies [21, 33–35, 38] relied on the Design, Implementation, Monitoring, and Evaluation Model when adapting the mhIST to other contexts, which uses qualitative data to inform item wording as well as translation and back-translation methods [43]. We reviewed back-translated versions from these five studies, a back-translated version adapted for use in Pakistan by Usman et al. [32], and one Spanish-language translation by Marsch et al. [31]. Consumer data from Lee et al. [21] were excluded at this stage because of substantive changes to the instrument for youth respondents. We combined item responses from all sites where items were an exact or near match into a single dataset for cross-site analysis.

##### Factor structure

We used exploratory factor analysis (EFA) to examine dimensionality and item loadings for each domain scale separately within the Consumer and Provider versions (rather than pooling all items within each version). EFA was guided by model fit statistics and parallel analysis using an oblique Geomin rotation in Mplus [44]. We expected factors identified during EFA of the Provider mhIST domain scales to align with subscales defined during scale development (Table S2). As no additional subscales were defined for Consumer mhIST, there were no pre-specified expectations for factor structure of Consumer scales.

We calculated Cronbach’s alpha (*α*) for each scale as measure of internal reliability. We also identified items for further review which had a high nonresponse rate (i.e., more than 20% of respondents selecting “Don’t know” or “Not applicable”), low covariance (< 0.1), cross-loading onto multiple factors, or a factor loading less than 0.4 [45]. Prior to cross-site analysis, we conducted EFA of each Consumer scale within each site, which informed methods used during cross-site analysis and are not presented here.

Where feasible, we drew a stratified random sample of two-thirds of respondents from each study site for cross-site EFA when examining Consumer mhIST scales and used the remaining third for validation [46]. The full sample was used, rather than a split sample validation approach, due to sample size constraints for the Provider mhIST and remaining Consumer scales. Results of the cross-site EFA informed the factor structure for the alignment analysis. If an item did not load onto a factor in the EFA, it was excluded from alignment.

##### Alignment

Lastly, we used alignment analysis to estimate measurement invariance and differential item functioning of Consumer scales; sample sizes were underpowered for alignment analysis of the Provider mhIST. Alignment analysis, a method of multi-group confirmatory factor analysis, allows researchers to estimate group-specific factor means and variances without requiring exact measurement invariance [47]. Asparouhov and Muthén developed the alignment method in response to practical limitations of conducting confirmatory factor with more than two groups, and their method simplifies and nearly automates measurement invariance analysis; a full description of the method is presented in their initial paper [46]. The method also produces an estimate of parameter invariance for model parameters in each group and can be used to evaluate the performance of a measure across multiple groups or settings. Another benefit of the alignment method is the use of pairwise—rather than listwise—comparison tests, i.e., responses from an individual are used even when some of the individual’s other response data are missing. As a result, the analysis is not impeded by missing or nonresponse data to the same extent as those relying on listwise comparisons.

We identified items for further review where alignment analysis indicated measurement noninvariance in factor loading or intercept across more than one site and for which the item-level noninvariance impacted variance in factor scores across sites. We also report the average item invariance for each scale using the *R*^2^ index, where 1 indicates full scalar invariance and 0 indicates full scalar noninvariance [47]. EFA and alignment analysis were used to examine dimensionality, factor structure, and measurement invariance of the mhIST. These results were then combined with a priori theory from the scale development process to comment on instrument performance and potential revisions. Alignment analysis was conducted in Mplus using Stata syntax; our syntax is presented in the supplementary materials (S4).

## Results

We compiled responses to the mhIST from *N =* 814 consumers and *N =* 223 providers of mental health interventions in six countries (Table 2). Average age across studies ranged from 35 to 55 among consumers and 28 to 44 among providers. Provider qualifications differed considerably across settings. Studies in Ukraine and Colombia primarily relied on formal health workers, while those in Myanmar, Thailand, Pakistan, and Zambia used briefly trained lay health workers or peers to deliver interventions.

### Consumer version

#### Exploratory factor analysis

We relied on model fit statistics, parallel analysis, and theory to guide model selection during exploratory factor analysis. We observed strong ceiling effects across participants in all studies during EFA, which reduced item variability and lead to low item discrimination and reduced utility of some model fit statistics (see Table S3; full item response distributions will be made available from the corresponding author upon request). Each scale of the Consumer mhIST was designed to measure a single implementation determinant or outcome. However, EFA results indicated only the Accessibility scale was unidimensional, where all but one item loaded onto a single factor for the Accessibility scale (Table 3). This non-loading item asked consumers whether they had a problem with the wait time before beginning the intervention (RA02) and has been reworded in subsequent use to not be reverse coded. Items on the Adoptability scale grouped around two factors: one focused on whether consumers had previously discussed the intervention with others, and another on the likelihood of consumers using the intervention in the future. One Adoptability item (AD06), which asked consumers whether they have encouraged others to seek out the intervention, cross-loaded onto both factors. Items on the Acceptability scale also loaded onto two distinct factors. The first focused on the experience of the consumer during the intervention, while the second factor focused on consumer perceptions of the provider (e.g., AC13: Did you feel that you could trust your counselor?). A single item (AC15), which asked consumers whether they understood the way things were explained during the intervention, did not load onto either factor.

**Table 3:** Structure of the Consumer mhIST scales

		Number of factors and subscale item loadings			
	*α*	1	2	3	x^a^
Adoptability (AD)	0.84	3, 4, 6	5–10		6
Acceptability (AC)	0.93	1–6	7–14		15
Appropriateness (AP)	0.90	1, 2, 5, 7	4, 9–14		3, 6, 8
Feasibility (FS)	0.86	1–5, 11, 12	6–10	13, 14	15
Accessibility (RA)	0.82	1, 3–8			2

*mhIST* Mental Health Implementation Science Tools

^a^Items not loading onto any factor at ≤ 0.4 or loading onto < 1 factor

EFA of the Appropriateness scale also indicated items loaded onto two factors: one related to intervention fit with culture and values, and another related to consumer perceptions of intervention effectiveness. Three items did not load onto either factor (AP03, AP06, and AP08). Lastly, the Feasibility scale was the only scale to have three factors identified in EFA and it also had the most variability in structure when comparing EFA results within and across sites. The three factors within the Feasibility scale focused on (1) consumer availability to engage in intervention components; (2) consumer resources for completing the intervention (e.g., funds for transportation); and (3) consumer perceptions of the location where the intervention was delivered. A single item about the ability of community members to seek out the intervention without stigma (FS15) did not load onto any factor.

#### Alignment

Results of the alignment analysis indicate the Consumer mhIST were relatively invariant across the six sites with consumer data, with an *R*^2^ ranging between 0.46 and 0.77 for each scale (Adoptability = 0.77, Acceptability = 0.65, Appropriateness = 0.69, Feasibility = 0.48, and Accessibility = 0.46). Most items were invariant across sites and only one item had noninvariant loadings in both loading and intercept across more than one site (AC12); this item asked consumers whether their provider was available when needed. Overall, several items from the Consumer mhIST were identified for further review due to nonresponse (25%), noninvariant loading (3%), or noninvariant intercept (7%); no items were found to have low covariance (Table 4).

**Table 4:** Items identified for further review from the Consumer mhIST

Item			EFA	Alignment	
Code	Label	Nonresponse ≥ 20%	Low or x-loading	Invariant loading	Invariant intercept
Adoptability					
AD06	Have encouraged		X		
Acceptability					
AC11	Provider interest	X			
AC12	Provider available			X	X
AC14	Provider qualified	X			
AC15	Understood program		X		
Appropriateness					
AP03	Fit schedule		X		
AP04	Fit males	X			
AP05	Fit females	X			
AP06	Organization good	X	X		
AP07	Good locale	X			
AP08	Address problems		X		
Feasibility					
FS06	Transport money	X			
FS07	Other money	X			
FS08	Maintain income	X			
FS11	Childcare available	X			
FS15	Seek without stigma		X		
Accessibility / Reach					
RA02	Wait time		X		
RA03	Most people seek	X			
RA05	Women seek	X			
RA06	Men seek	X			
RA07	Caregivers seek	X			
RA08	Children seek	X			

*mhIST* Mental Health Implementation Science Tools, *EFA* Exploratory factor analysis, *x-loading* cross-loading

### Provider version

The total number of providers from each site is much smaller than the number of consumers, ranging in sample size from 10 to 34 in five out of six studies where provider data was available. A notable exception is the trial of trauma-focused cognitive behavioral therapy in Zambia [35], where 101 providers completed the mhIST. Small provider sample sizes precluded cross-site alignment analysis. However, EFA of provider data from all sites combined yielded informative results. We identified between one to four factors for each of the Provider scales (Table 5).

**Table 5:** Structure of Provider mhIST scales

		Factors and item loadings				
	*α*	1	2	3	4	x^a^
Adoptability (AD)	0.72	1, 5	2–6	6, 8, 9		6, 7
Acceptability (AC)	0.81	1–6, 8, 10, 12	7, 11, 13			9
Appropriateness (AP)	0.84	1–3	4, 5	1, 2, 7–10	11–16	
Feasibility (FS)	0.83	1–5, 13	7–12, 14, 15			
Accessibility (RA)	0.79	3–7				9
Organizational Climate (OC)	0.90	1, 3–10, 12	7, 11, 13–18			2, 7
General Leadership (GL)	0.87	1–9				

*mhIST* Mental Health Implementation Science Tools

^a^Items not loading onto any factor at ≤ 0.4 or cross-loading onto < 1 factor

Factors identified through EFA generally aligned with a priori subscales of the Provider mhIST (Table S2), with the exception of the Adoptability scale. Despite no predefined subscales, EFA indicated items in the Adoptability scale grouped around the following themes: (1) past discussions about program within the organization, (2) past discussions outside the organization, and (3) future program provision. Factors of the Acceptability scale generally aligned with the two subscales “Program/Treatment” and “Individual Professionalism.” The Appropriateness scale appeared to have two factors within the “Social/Cultural” subscale, while the remaining factors generally align with the subscales of “Self Perception of Effectiveness” and “Task Fit.” Rather than the four subscales defined by the scale developers, EFA indicated the Feasibility scale had only two factors: one relating to provider time and another to provider support and resources. The Accessibility scale, which has no predefined subscales, performed unidimensionally.

The mhIST developers included two additional scales in the Provider version not present in the Consumer, Organizational Climate (OC) and General Leadership (GL). The factor structure of the Organizational Climate scale generally aligns with the two predefined subscales: “Personal Feelings at Work” and “Perceived Work Environment.” The General Leadership scale performed unidimensionally, with all items loading on a single factor. Several items were identified for review during cross-site analysis due to nonresponse (19%) or low factor loading (7%); no items had low covariance (Table 6).

**Table 6:** Items identified for further review from the Provider mhIST

Item			EFA
Code	Label	Nonresponse ≥ 20%	Low or x-loading
Adoptability			
AD03	Discussed impact	X	
AD04	Discussed experience	X	
AD06	Have encouraged		X
AD07	Refer others		X
Acceptability			
AC09	Provider abilities		X
Appropriateness			
AP06	Military culture	X	
Feasibility			
FS11	Program budget	X	
FS12	Enough providers	X	
Accessibility			
RA01	Community aware	X	
RA02	Wait time	X	
RA07	Caregivers seek	X	
RA09	Military seek	X	X
Organizational Climate			
OC02	Workload OK		X
OC07	Org prof. growth		X
OC17	Program fit in org	X	
OC18	Program useful to org	X	
General Leadership			
GL09	Leaders strategies	X	

*mhIST* Mental Health Implementation Science Tools, *EFA* Exploratory factor analysis, *x-loading* cross-loading

## Discussion

Our study is the first to evaluate the psychometric performance of implementation measures for mental health across several countries. We sought to build the evidence base for and inform the use of the Mental Health Implementation Science Tools (mhIST) across diverse contexts and populations by analyzing responses from 814 consumers and 223 providers of mental health interventions across six countries. Our results point to measure structure and item performance of the Consumer and Provider versions of the mhIST. These findings provide evidence of the internal validity and reliability of the tools in diverse settings and highlights areas for further scale refinement through item-level review and subscale revisions.

We found evidence for good to excellent internal reliability of the Consumer mhIST, with Cronbach’s *α* greater than 0.8 for all scales. However, factor analysis indicated four of five scales are multidimensional, in which case Cronbach’s *α* may not be the best indicator of reliability, despite a high *α* [48]. We also provide evidence the Consumer scales measure the same constructs across populations and settings; alignment analysis indicated acceptable levels of invariance for all five measures, though there was considerable range in the invariance index [47, 49]. Lastly, we highlighted items across all scales that merit further review and potential revision due to participant nonresponse, low factor loadings, or noninvariance; no item meet the criterion for low covariance. Our results can inform future iterations of the mhIST for an enhanced valid and reliable cross-cultural set of measures.

We found scales within the Provider mhIST to have similar internal reliability to those within the Consumer version, with an *α* of at least 0.8 for all Provider scales except Adoptability. In contrast to the Consumer version, several of the Provider scales were defined to have subscales during scale development. Results indicated five of eight scales were multidimensional during factor analysis, with dimensionality generally aligning with predefined subscales. Unfortunately, the small number of providers from each study did not allow for adequately powered alignment analysis. Measurement invariance of the Provider scales is therefore relatively unknown. Only six items were identified for review and potential revision due to low or cross item loading during EFA.

These findings build on the research of Haroz et al. [18] to provide additional evidence of reliability and validity for a set of implementation measures developed specifically for mental health interventions in LMIC. Estimates of internal consistency for the mhIST were good to excellent for nearly all scales, comparable to estimates reported by Haroz et al. [18], and greater than internal consistency estimates from most measures identified in a review of implementation science measures by Clinton-McHarg et al. [13]. Internal consistency estimates for the Acceptability, Appropriateness, and Feasibility Provider scales were slightly lower than those reported for the alternative Acceptability, Appropriateness, and Feasibility measures developed by Weiner et al. [50], though both sets of measures have demonstrated excellent psychometric properties and have distinct advantages and disadvantages. The scales developed by Weiner and colleagues are short, unidimensional, and efficient measures of their respective implementation outcomes, though their simplicity also means they carry limited explanatory power. Despite a favorable rating for usability, a 2020 review also rated the reliability and validity of these scales poorly due to the lack of evidence for certain types of reliability and validity not reported by Weiner et al. [50], such as structural validity [51]. In addition, translation of the subtle English synonyms used in these scales may be challenging or impossible in diverse global contexts. The mhIST scales are longer, multidimensional, and less efficient than scales developed by Weiner and colleagues, though their complexity derives from their explanatory power. In fact, individual mhIST items can be helpful to guide programmatic needs and adaptations, independent of scale summary scores.

While our work identified items for revision or removal based on psychometric properties, there may be other reasons to retain items. We observed strong ceiling effects and low item variability across participants in the six studies. Despite this, items with ceiling effects are informative for implementation outcomes when even small deviations from favorable responses are informative, e.g., it is highly relevant to programs whether all participants report whether counseling spaces were confidential (item FS14). From this perspective, the value of several items is not necessarily differentiation but information or confirmation. Moreover, items with low or cross factor loadings may still have individual utility despite not aligning within the scale or subscale for which it was developed. For example, an item asking whether consumers understood the way in which things were explained may still provide useful program information despite not sufficiently loading onto any factor in the Acceptability scale. It may be preferable to retain and separate items with low or cross loadings, rather than removing them completely, since each scale is intended to be averaged to provide a summary score for the given implementation outcome or determinant. Other measures in the literature may not capture the breath of potential items related to an implementation domain [12]. While this approach to measurement has benefits for psychometrics, ultimately it may not be that informative for implementation studies. The ability of the mhIST to both be scored as scales representative of an underlying construct, and to track item-level indicators of implementation, enables flexibility for research and practice use.

### Limitations

Our primary limitation was relatively small sample sizes for psychometric analysis despite pooling data from several contexts and studies. Most notably, we were unable to evaluate measurement invariance of the Provider mhIST due to the small number of intervention providers within each contributing study, except for the trial of trauma-informed cognitive behavioral therapy in Zambia [35]. Small sample sizes precluded both within-site EFA and cross-site alignment analysis, which would provide information on how measure structure and item performance may differ across the six settings. The large sample of providers from Zambia relative to other settings also disproportionately weights results to responses from Zambian providers in the EFA of pooled Provider mhIST data, i.e., these results are more representative of Zambian providers than those from other settings. Factor structures of the Provider tools presented should be considered informative but not conclusive given results are based on a relatively low ratio of observations to items during EFA. Small sample sizes also precluded cross-validation methods to prevent overfitting of the alignment analysis. Nonresponse was high for some items. However, since the aim of the study is a pragmatic evaluation of measure performance, nonresponse is in fact informative and useful when considering how the instrument may be improved. We also mitigate the impact of missing data in our analysis by use of pairwise rather than listwise comparison in EFA and alignment analysis, allowing for the inclusions of observations with some data missing. Finally, while all data came from consumers or providers of mental health services in LMIC, the data may have differed on other important factors that may contribute to cross-site comparison (e.g., type of mental health intervention, demographics of the samples). Due to sample sizes, we were unable to control for these potential confounders.

Findings from our study should be interpreted alongside a few considerations. The first is the need to balance theory- and data-driven approaches within psychometric research. The mhIST took over 5 years to develop and was based on leading theoretical frameworks and existing measures, expert consultation, and iterative pilot tests. Data from the seven studies included in this analysis indicate how the instrument performed across diverse settings. Nonetheless, results should not be considered prescriptive. For instance, removing an item may lead to modest improvements in internal reliability or measurement invariance, though this evidence should be weighed alongside a priori theory and reasoning. Second, the scope of the present study does not include all relevant types of validity and reliability, such as test-retest reliability, criterion validity, or predictive validity. Further research in these areas will become feasible as the mhIST are more widely adopted within global mental health research. The development of a centralized repository would help to standardize and facilitate future psychometric research of these and other implementation measures. Lastly and relatedly, our findings are limited by the lack of gold standard validation approaches within implementation research. While some studies have used vignette-based approaches [18, 50], in general measurement in implementation research is impeded by the complexity of defining and operationalizing major domains of implementation science. There is a need to establish best practices for validation techniques for implementation research in mental health, particularly within LMIC. These best practices should be pragmatic, consensus-based guidelines for selecting appropriate implementation measures depending on use case and how to adapt and validate measures in new contexts, including whether and the extent to which formal validation is necessary.

## Conclusion

We found the Consumer mhIST performed similarly across diverse populations and contexts within LMIC and provide psychometric evidence of item performance and measure structure for the Consumer and Provider versions. Our findings will ultimately inform a future iteration of the mhIST that is based on retaining items robust across settings and serves as a valid and reliable tool for implementation research in mental health within LMIC.

## Supplementary Information

**Additional file 1: Table S1.** Mental Health Implementation Science Tools (mhIST), Consumer version. **Table S2.** Mental Health Implementation Science Tools (mhIST), Provider version. **Table S3.** Fit statistics for models selected in exploratory factor analysis. **S4.** Stata syntax for alignment analysis.

## Data Availability

The data that support the findings of this study are available from corresponding authors of each contributing study but restrictions apply to the availability of these data, which were used under agreement for the current study, and so are not publicly available. Data are however available from the authors upon reasonable request and with permission from corresponding authors of contributing studies.

## Abbreviations

AC: Acceptability
AD: Adoptability
AUD: Alcohol use disorder
AP: Appropriateness
BA: Behavioral activation
CBT: Cognitive behavioral therapy
CETA: Common Elements Treatment Approach
EFA: Exploratory factor analysis
FS: Feasibility
GL: General Leadership
IDP: Internally displaced persons
IPV: Interpersonal violence
OC: Organizational Climate
LMIC: Low- and middle-income countries
mhIST: Mental Health Implementation Science Tools
PST: Problem solving therapy
RA: Accessibility
SD: Standard deviation
TF-CBT: Trauma-focused cognitive behavioral therapy
