# Supplementary material for: Psychometric performance of the Mental Health Implementation Science Tools (mhIST) across six low- and middle-income countries
Source: Implement Sci Commun. 2022 May 19;3:54. doi: 10.1186/s43058-022-00301-6 (PMC9118868; doi:10.1186/s43058-022-00301-6)
Supplement: Supplementary file 1 — Additional file 1: Table S1. Mental Health Implementation Science Tools (mhIST), Consumer version. Table S2. Mental Health Implementation Science Tools (mhIST), Provider version. Table S3. Fit statistics for models selected in exploratory factor analysis. S4. Stata syntax for alignment analysis. [file 43058_2022_301_MOESM1_ESM.docx]

**SUPPLEMENTARY MATERIALS**

**Table S1.** Mental Health Implementation Science Tools (mhIST), Consumer version

| Code | Label | Item | |
| --- | --- | --- | --- |
| ***ADOPTION*** | |  | |
| AD01* | Why start | Why did you start THE PROGRAM? | |
| AD02* | Why end | Why did you stop THE PROGRAM? | |
| AD03 | Discussed program | Have you discussed with others (e.g., family, friends, coworkers, or any other people) what THE PROGRAM is? | |
| AD04 | Discussed participation | Have you discussed with others (e.g., family, friends, coworkers, or any other people) what you need to do if you participate in THE PROGRAM? | |
| AD05 | Have used skills | Have you used the skills you learned in THE PROGRAM? | |
| AD06 | Have encouraged | Have you encouraged others to seek THE PROGRAM services? | |
| AD07 | Refer others | Would you refer others with similar problems to THE PROGRAM? | |
| AD08 | Return to program | Would you return to THE PROGRAM services if you felt like you needed them in the future? | |
| AD09 | Will use skills | Would you continue to use the skills you learned in THE PROGRAM? | |
| AD10 | Will encourage | Would you encourage others to seek THE PROGRAM services? | |
|  |  |  | |
| ***ACCEPTABILITY*** | |  | |
| AC01 | Overall like | Overall, did you like THE PROGRAM? | |
| AC02 | Like attending | Did you like attending THE PROGRAM sessions? | |
| AC03 | Satisfied | Did you feel satisfied with THE PROGRAM services received? | |
| AC04 | Enjoy learning | Did you enjoy learning THE PROGRAM? | |
| AC05 | Useful skills | Do you feel that the skills you learned in THE PROGRAM are useful? | |
| AC06 | Makes sense | Do you feel that the components of THE PROGRAM make sense to you? | |
| AC07 | Ask questions | Did you feel comfortable raising questions to your counselor? | |
| AC08 | Provider listened | Did you feel that your counselor listened to your concerns and questions about THE PROGRAM? | |
| AC09 | Provider abilities | Did you feel satisfied with your counselor’s abilities in THE PROGRAM? | |
| AC10 | Questions answered | Did you feel that your counselor addressed any questions or concerns you had about THE PROGRAM? | |
| AC11 | Provider interest | Did your counselor take an interest in you? (UKV) | |
| AC12 | Provider available | Was your counselor available when you needed to talk to him/her? | |
| AC13 | Trust provider | Did you feel that you could trust your counselor? | |
| AC14 | Provider qualified | Did you feel that your provider was qualified enough to deliver THE PROGRAM? (UK, UZ) | |
| AC15 | Understood program | Did you feel that you understood the way things were explained to you during THE PROGRAM? | |
|  |  |  | |
| ***APPROPRIATENESS*** | |  | |
| AP01 | Cultural values | Does THE PROGRAM fit with your cultural values? | |
| AP02 | Personal values | Does THE PROGRAM fit with your personal values? | |
| AP03 | Fit schedule | Did participating in THE PROGRAM fit into your daily schedule and routine? | |
| AP04 | Fit males | Does THE PROGRAM fit with the male culture in your country? | |
| AP05 | Fit females | Does THE PROGRAM fit with the female culture in your country? | |
| AP07 | Organization good | Is the organization where your counselor works a good place for delivery of THE PROGRAM? | |
| AP08 | Locale good | Did you feel comfortable with the location where you usually met your counselor? | |
| AP09 | Address problems | Do you believe THE PROGRAM was a good way to address your problems? | |
| AP10 | Helpful strategies | Did you learn helpful strategies in THE PROGRAM to deal with your problematic thoughts, feelings and behaviors? | |
| AP11 | Helped problems | Do you think THE PROGRAM helped with you your problems? | |
| AP12 | Help others | Do you believe THE PROGRAM is appropriate for helping other people with similar problems as yours? | |
| AP13 | Common problems | Do you believe THE PROGRAM addresses problems that are common in your community? | |
| AP14 | Relevant to others | Do you believe the skills taught in THE PROGRAM would be relevant to other people like yourself? | |
|  |  |  | |
| ***FEASIBILITY*** | |  | |
| FP01 | Attendance | Have you been able to attend the weekly sessions of THE PROGRAM without difficulty? | |
| FP02 | Flexibility | Were THE PROGRAM sessions scheduled with enough flexibility to meet your needs? | |
| FP03 | Timeliness | Was your counselor on time for your sessions? | |
| FP04 | Away from duties | Was it easy for you to get away from your duties (eg. work, parenting) to attend THE PROGRAM? | |
| FP05 | Time spent | Was the amount of time you spent doing THE PROGRAM homework each week manageable? | |
| FP06 | Transport money | Did you have enough money to pay for transport to get to THE PROGRAM sessions? | |
| FP07 | Other money | Did you have enough money to pay for any other things you needed to get to THE PROGRAM sessions? | |
| FP08 | Maintain income | Were you able to maintain your regular income level while receiving THE PROGRAM? | |
| FP09 | Enough resources | Did you have enough resources (phone, talk time) to communicate with your counselor when needed? | |
| FP10 | Emotional support | Did you have the emotional support that you needed from your family and friends to attend THE PROGRAM? | |
| FP11 | Childcare | Were you able to find childcare when you needed it to attend THE PROGRAM? | |
| FP12 | Safe travel | In general, did you feel safe to travel to weekly THE PROGRAM sessions? | |
| FP13 | Safe locale | Did you feel the place you met with your counselor was safe? | |
| FP14 | Private locale | Did you feel the place you met with your counselor was private and confidential? | |
| FP15 | Stigma | Do you believe people in your community could seek help for mental health problems from THE PROGRAM without fear of how others would view them? | |
|  |  |  | |
| ***ACCESSIBILITY / REACH*** | |  | |
| RA01 | People aware | Are people in the community aware that THE PROGRAM services are available? | |
| RA02 | Wait time | When you sought help, how much of a problem did you have with the amount of time you had to wait to begin THE PROGRAM? | |
| RA03 | Most people seek | Would most people in the community who need mental health services seek THE PROGRAM services? | |
| RA04 | Poorest seek | Would the poorest people in the community who need mental health services seek THE PROGRAM services? | |
| RA05 | Women seek | Would women who need mental health services seek THE PROGRAM services? | |
| RA06 | Men seek | Would men who need mental health services seek THE PROGRAM services? | |
| RA07 | Caregivers seek | Would parents or other caretakers seek THE PROGRAM services if their children needed it? | |
| RA08 | Children seek | Would children without parents seek THE PROGRAM services if needed? | |
| RA09 | Military seek | Would people in the military seek THE PROGRAM services? (Ukraine only) | |
| RA10* | Who use | Who in your community would not seek THE PROGRAM services if needed? | |
| *items not included in the analysis due to non-Likert response type | | |  |

**Table S2.** Mental Health Implementation Science Tools (mhIST), Provider version

| Code | Label | Item |
| --- | --- | --- |
| **ADOPTION** |  |  |
| AD01 | Discussed provision | Have you discussed with other providers and staff about what is needed to continue to provide THE PROGRAM in the future? |
| AD02 | Discussed general | Have you discussed with others (e.g. family, friends, coworkers, or any other people) what THE PROGRAM is in general terms? |
| AD03 | Discussed impact | Have you discussed with others (e.g. family, friends, coworkers, or any other people) THE PROGRAM's impact on clients? |
| AD04 | Discussed experience | Have you discussed with others (e.g. family, friends, coworkers, or any other people) your experiences as a THE PROGRAM provider? |
| AD05 | Encouraged providers | Have you encouraged others outside your organization to become a provider of THE PROGRAM? |
| AD06 | Continue providing | Will you continue to provide THE PROGRAM in the future? |
| AD07 | Seek supervision | Will you seek regular supervision for THE PROGRAM in the future? |
| AD08 | Use paperwork | Will you continue to track clients' progress using appropriate paperwork (i.e. assessments, weekly monitoring forms)? |
| AD09 | High priority | Will providing THE PROGRAM be a high priority for you in the future? |
|  |  |  |
| **ACCEPTABILITY** | |  |
| *Program Treatment* | |  |
| AC01 | Like providing | Do you like providing THE PROGRAM? |
| AC02 | Feel good | Do you feel good about providing THE PROGRAM? |
| AC03 | Good treatment | Do you feel good about THE PROGRAM as a treatment for client's mental health problems? |
| AC04 | Enjoy learning | Did you enjoy learning THE PROGRAM? |
| AC05 | Useful skill | Do you feel that the skills you have learned by providing this service will be useful in helping clients? |
| AC06 | Makes sense | Do you feel that the components (e.g. the activities that you do in sessions with clients) of THE PROGRAM make sense? |
| AC07 | Satisfied training | How satisfied are you with the training you received in THE PROGRAM? |
| AC08 | Satisfied supervision | How satisfied are you with the supervision you received when providing THE PROGRAM? |
| AC09 | Language clear | Is THE PROGRAM material (i.e. manual, paperwork) clear in LOCAL LANGUAGE? |
| *Individual Professionalism* | |  |
| AC10 | Feel success | Does being a provider of THE PROGRAM help you feel successful in your job? |
| AC11 | Job stability | Does being a provider of THE PROGRAM allow you to have job stability? |
| AC12 | Professional goals | Is providing THE PROGRAM consistent with you professional goals? |
| AC13 | Job advancement | Does being a provider of THE PROGRAM create more opportunities for your career advancement? |
|  |  |  |
| **APPROPRIATENESS** | |  |
| *Socio/Cultural* | |  |
| AP01 | Client cultural values | How well does THE PROGRAM fit with the cultural values of your clients? |
| AP02 | Personal values | How well does THE PROGRAM fit with your own personal values? |
| AP03 | Your cultural values | How well does THE PROGRAM fit with your cultural values? |
| AP04 | Male culture | Is THE PROGRAM consistent with the male culture in your country? |
| AP05 | Female culture | Is THE PROGRAM consistent with the female culture in your country? |
| AP06 | Military culture | Is THE PROGRAM consistent with the military culture in your country? |
| *Self-Perception of Effectiveness* | |  |
| AP07 | Address client issues | Is THE PROGRAM a good way to address your clients' problems? |
| AP08 | Clients feel better | Does THE PROGRAM help your clients feel better? |
| AP09 | Effective treatment | Is THE PROGRAM effective for your clients' mental health problems? |
| AP10 | Effective others | Is THE PROGRAM likely to be effective for people in other parts of your country? |
| *Task Fit* |  |  |
| AP11 | Program as job | Is providing THE PROGRAM something you feel you should be doing as part of your job? |
| AP12 | Supervision as job | Is participating in supervision for THE PROGRAM something you feel you should be doing as part of your job? |
| AP13 | Paperwork as job | Is tracking client progress through paperwork for THE PROGRAM something you feel you should be doing as part of your job? |
| AP14 | Colleague support | Is providing THE PROGRAM something that your colleagues support you to do as part of your job? |
| AP15 | Program as JD | Does provision of THE PROGRAM fit with your current job description? |
| AP16 | Supervision as JD | Does participating in the supervision for THE PRROGRAM fit with your current job description? |
|  |  |  |
| **FEASIBILITY** | |  |
| *Skills* |  |  |
| FS01 | Sufficient skills | Are you sufficiently skilled at providing THE PROGRAM to your clients? |
| *Time* |  |  |
| FS02 | All program time | Do you have enough time for all the activities that go into providing THE PROGRAM (eg: documentation, handling, safety issues, etc.)? |
| FS03 | Supervision time | Do you have enough time to spend in supervision activities related to THE PROGRAM? |
| FS04 | Time for provision | Do you have enough time to regularly provide THE PROGRAM to those who need it? |
| FS05 | Time for travel | Do you have enough time to travel to and from appointments for THE PROGRAM? |
| *Resources* |  |  |
| FS06 | Paid enough | Are you paid enough to provide THE PROGRAM? |
| FS07 | Transport funds | Are you provided with the necessary money for transportation to regularly provide THE PROGRAM? |
| FS08 | Right equipment | Do you have the right equipment (eg: pens/pencils/computer/internet, toys, art supplies) to regularly provide THE PROGRAM? |
| FS09 | Phone talk time | Do you have the resources (eg: phone, talk time) to reach your clients and/or supervisor when needed? |
| FS10 | Private space | Do you have sufficient access to private space to meet with clients in private? |
| FS11 | Program budget | Does the overall program have sufficient budget to provide THE PROGRAM as needed? |
| FS12 | Enough providers | Are there enough providers trained in THE PROGRAM for those that need it within the population you work with? |
| FS13 | Reach supervisor | Are you able to reach your supervisor when needed? |
| FS14 | Clinical support | Do you have sufficient access to continued clinical support and training? |
| FS15 | Self-care | Do you have support for self-care when needed? |
| FS16 | Emotional support | Do you feel that you have access to the support you need to be emotionally healthy enough to provide THE PROGRAM? |
| FS17* | Hours per week | In general, about how many hours per week do you spend providing THE PROGRAM to clients? |
| FS18* | Days per week | In general, about how many days per week are you available to provide THE PROGRAM to clients? |
| FS19* | Other hours | How many hours a week do you spend on other activities apart from seeing clients and supervision, related to providing THE PROGRAM? |
| FS20* | Supervision hours | How many hours per week do you spend in supervision related meetings? |
|  |  |  |
| **ACCESSIBILITY / REACH** | |  |
| RA01 | Community aware | Are people in the community aware that THE PROGRAM is available? |
| RA02 | Wait time | When clients seek help, how much of a problem is the amount of time they have to wait to begin THE PROGRAM? |
| RA03 | Most people seek | Would most people in your community who need them, seek THE PROGRAM? |
| RA04 | Poorest seek | Would the poorest people in your community who need them, seek THE PROGRAM services? |
| RA05 | Women seek | Would women who need them seek THE PROGRAM services? |
| RA06 | Men seek | Would men who need them seek THE PROGRAM services? |
| RA07 | Caregivers seek | Would parents or other caretakers seek THE PROGRAM services for their children if needed? |
| RA08 | Children seek | Would children without parents or other caregivers seek THE PROGRAM services if needed? |
| RA09 | Military seek | Would people in the military seek THE PROGRAM services if needed? |
| RA10* | Not seek | Who would not seek THE PROGRAM services within your community? |
|  |  |  |
| **ORGANIZATIONAL CLIMATE** | |  |
| *Personal Feelings at Work* | |  |
| OC01 | Your morale | Is your morale at work be high? |
| OC02 | Workload OK | Is your workload manageable (meaning you do not feel stressed or overworked)? |
| OC03 | Informed about org | Do you feel you are well informed on things you should know about within your organization? |
| OC04 | Informed within org | Does your organization keep you well informed on things you should know about within the organization? |
| OC05 | Who to go to | When you have a problem at work, you know who to go to for the answer? |
| OC06 | Responsive | When you have a problem, the person you go is responsive? |
| OC07 | Org prof growth | Does the organization promote your professional growth? |
| OC08 | Org adaptable | Is the organization adaptable to your needs? |
| OC09 | Paid on time | Are you regularly paid on time? |
| OC10 | Job pay | Are you satisfied with the monetary compensation you receive for your job? |
| OC11 | Care about org | Do you care about the direction and success of the larger organization that is implementing THE PROGRAM? |
| OC12 | Learning opportunities | Are there enough learning opportunities offered to you through the organization? |
| *Perceived Work Environment* | |  |
| OC13 | Good communication | To what extent is the communication between people at the organization positive and useful? |
| OC14 | Positive place | Does the place where you work feel like a positive work environment to work and learn in? |
| OC15 | Staff cohesion | Do you feel that there is a high level of cohesion within your workplace (e.g staff getting along, working together as a team)? |
| OC16 | Staff morale | Does staff at your workplace have a high amount of morale? |
| OC17 | Program fit in org | Do you think THE PROGRAM fits with the goals of your organization? |
| OC18 | Program useful to org | Do you think providing THE PROGRAM is useful for your organization? |
|  |  |  |
| **LEADERSHIP IN IMPLEMENTATION** | | |
| *Proactive Leadership* | |  |
| LI01 | Standards for imp | Do the leaders at your organization have clear quality standards for implementation of THE PROGRAM? |
| LI02 | Facilitate imp | Do the leaders at your organization have a plan to facilitate implementation of THE PROGRAM? |
| LI03 | Remove obstacles | Do the leaders at your organization remove obstacles to implementation of THE PROGRAM? |
| *Knowledgeable Leadership* | |  |
| IL04 | Leaders know | Do the leaders at your organization know what they are talking about when it comes to THE PROGRAM? |
| IL05 | Org know | Are the leaders at your organization knowledgeable about THE PROGRAM? |
| IL06 | Staff know | Are the leaders at your organization able to answer staff questions about THE PROGRAM? |
| *Supportive Leadership* | |  |
| LI07 | Support use | Do the leaders at your organization support provider efforts to use THE PROGRAM? |
| LI08 | Support learning | Do the leaders at your organization support provider efforts to learn more about THE PROGRAM? |
| LI09 | Efforts appreciated | Do the leaders at your organization recognize and appreciate provider efforts? |
| *Perseverant Leadership* | |  |
| IL10 | Leaders persevere | Do the leaders at your organization persevere through the ups and downs of implementing THE PROGRAM? |
| IL11 | Leaders work through | Do the leaders at your organization continue to work through the challenges of implementing THE PROGRAM? |
| IL12 | Leaders address | Do the leaders at your organization effectively address critical issues regarding implementation of THE PROGRAM? |
|  |  |  |
| **GENERAL LEADERSHIP SKILLS** | |  |
| GL01 | Leaders good | Are the leaders at your organization good leaders? |
| GL02 | Leaders approachable | Are the leaders at your organization easy to talk to? |
| GL03 | Leaders trustworthy | Are the leaders at your organization trustworthy? |
| GL04 | Respect leaders | Do the leaders at your organization behave in ways that gain respect from staff members? |
| GL05 | Decisions of leaders | Do the leaders at your organization make appropriate decisions to help ensure the success of THE PROGRAM? |
| GL06 | Leaders open | Are the leaders at your organization open to hearing ideas that differ from their own? |
| GL07 | Leaders share goals | Do the leaders at your organization help staff members see how program goals align with their personal goals? |
| GL08 | Leaders share future | Do the leaders at your organization talk about goals for the future for the program? |
| GL09 | Leaders strategies | Do the leaders at your organization have strategies in place to help achieve program goals? |
| *items not included in the analysis due to non-Likert response type | | |

| **Table S3.** Fit statistics for models selected in exploratory factor analysis | | | |
| --- | --- | --- | --- |
|  | CFI | TLI | RMSEA |
| Consumer Scales | | | |
| Adoption | 0.907 | 0.800 | 0.150 |
| Acceptability | 0.929 | 0.902 | 0.094 |
| Appropriateness | 0.903 | 0.857 | 0.098 |
| Feasibility | 0.918 | 0.864 | 0.070 |
| Accessibility / Reach | 0.987 | 0.982 | 0.047 |
| Provider Scales | | | |
| Adoption | 0.992 | 0.975 | 0.038 |
| Acceptability | 0.982 | 0.974 | 0.046 |
| Appropriateness | 0.977 | 0.956 | 0.620 |
| Feasibility | 0.968 | 0.956 | 0.980 |
| Accessibility / Reach | 0.996 | 0.995 | 0.037 |
| Organizational Climate | 0.935 | 0.916 | 0.094 |
| General Leadership | 0.952 | 0.936 | 0.144 |
| CFL comparative fit index, TLI Tucker-Lewis index, RMSEA root mean square error of approximation | | | |
|  |  |  |  |

S4. Stata syntax for alignment analysis

**********************************

*** mhIST ALIGNMENT ANALYSIS ***

*** CLIENT LEVEL ***

**********************************

* call in data

use “~/D&I Measurement Supplement/7. Data from U19s/Combined data/ClientDI_01Apr20_combined.dta"

cd "~/Dropbox/D&I Measurement Supplement/7. Data from U19s/Combined data"

*** 1. ADOPTION MEASURE

*** 1a. Re-check EFA

runmplus adopt3-adopt10, ///

variable( ///

usevar = adopt3-adopt10; ///

) analysis( ///

type = EFA 1 4; ///

rotation = geomin; ///

!parallel = 50; ///

) output(modindices;)

*** 1b. Alignment

runmplus adopt3-adopt10 country, ///

coverage(0) varnocheck ///

///cat(s01_1-s14_1 s38_1 s39_1 s41_1 s51_1) ///

variable( ///

classes=d(5); ///

knownclass=d(country); ///

) analysis( ///

type=mixture; ///

estimator=bayes; ///

!fbiter=1000; thin=40; ///

!chains=1; ///

alignment = free ; /// this makes the lv to N(0,1) in a reference dataset.

algorithm=integration; ///

/// process=2; ///

) model( ///

%OVERALL% ///

AD1 by adopt3 adopt4 adopt6; ///

AD2 by adopt5 adopt7 adopt8 adopt9 adopt10; ///

) output(stdyx; align; modindices;) ///

savedata(save=fscores(10); file=fscores_dep1_pv.txt)

*** 2. ACCEPTABILITY MEASURE

*** 2a. Re-check PCA / EFA

pca accept1-accept15

fapara, pca reps(20)

// pca indicates 2 factors

factor adopt3-adopt10, ipf factor(2)

rotate, promax blanks(.32)

estat common

runmplus accept1-accept15 country, ///

variable( ///

usevar = accept1-accept15; ///

) analysis( ///

type = EFA 1 4; ///

rotation = geomin; ///

parallel = 50; ///

) output(modindices;)

// parallel & model fit stats indicatee 2-factor model

*** 2b. Alignment

runmplus accept1-accept15 country, ///

coverage(0) varnocheck ///

///cat(s01_1-s14_1 s38_1 s39_1 s41_1 s51_1) ///

variable( ///

classes=d(5); ///

knownclass=d(country); ///

) analysis( ///

type=mixture; ///

estimator=bayes; ///

!fbiter=1000; thin=40; ///

!chains=1; ///

alignment = free ; /// this makes the lv to N(0,1) in a reference dataset.

algorithm=integration; ///

/// process=2; ///

) model( ///

%OVERALL% ///

AC1 by accept1-accept6; ///

AC2 by accept7-accept14; ///

/// dep by s01_1* s02_1-s14_1 s38_1 s39_1 s41_1 s51_1; dep@1; ) ///

) output(stdyx; align; modindices;) ///

savedata(save=fscores(10); file=fscores_dep1_pv.txt)

*** 3. APPROPRIATENESS MEASURE

*** 2a. Re-check PCA / EFA

pca approp1-approp5 approp7-approp14

fapara, pca reps(20)

// pca indicates 2 factors

factor approp1-approp5 approp7-approp14, ipf factor(2)

rotate, promax blanks(.32)

estat common

runmplus approp1-approp5 approp7-approp14 country, ///

variable( ///

usevar = approp1-approp5 approp7-approp14; ///

) analysis( ///

type = EFA 1 4; ///

rotation = geomin; ///

parallel = 50; ///

) output(modindices;)

// parallel & model fit stats indicate a 2-factor model

*** 3b. Alignment

runmplus approp1-approp5 approp7-approp14 country, ///

coverage(0) varnocheck ///

///cat() ///

variable( ///

classes=d(5); ///

knownclass=d(country); ///

) analysis( ///

type=mixture; ///

estimator=bayes; ///

fbiter=1000; ///

thin=40; ///

!chains=1; ///

alignment = free ; /// this makes the lv to N(0,1) in a reference dataset.

algorithm=integration; ///

/// process=2; ///

) model( ///

%OVERALL% ///

AP1 by approp1 approp2 approp5 approp7; ///

AP2 by approp4 approp9-approp14; ///

/// dep by s01_1* s02_1-s14_1 s38_1 s39_1 s41_1 s51_1; dep@1; ) ///

) output(stdyx; align; modindices;) ///

savedata(save=fscores(10); file=fscores_dep1_pv.txt)

*** 4. FEASIBILITY MEASURE

*** 4a. Re-check PCA / EFA

// See Analysis report

runmplus feas1-feas15, ///

variable( ///

usevar = feas1-feas15; ///

) analysis( ///

type = EFA 1 6; ///

rotation = geomin; ///

parallel = 50; ///

) output(modindices;)

*** 4b. Alignment

runmplus feas1-feas14 country, ///

coverage(0) varnocheck ///

///cat() ///

variable( ///

classes=d(5); ///

knownclass=d(country); ///

) analysis( ///

type=mixture; ///

estimator=bayes; ///

fbiter=1000; ///

thin=40; ///

!chains=1; ///

alignment = free ; /// this makes the lv to N(0,1) in a reference dataset.

algorithm=integration; ///

) model( ///

%OVERALL% ///

FS1 by feas1-feas5 feas11 feas12 ; ///

FS2 by feas6-feas10; ///

FS3 by feas13 feas14; ///

) output( align; ) ///stdyx;modindices;

savedata(save=fscores(10); file=fscores_dep1_pv.txt)

*** 5. ACCESSIBILITY MEASURE

*** 5a. Re-check PCA / EFA

runmplus access1-access8 country, ///

variable( ///

usevar = access1-access8; ///

) analysis( ///

type = EFA 1 4; ///

rotation = geomin; ///

parallel = 50; ///

) output(modindices;)

// 1-factor model indicated

*** 5b. Alignment

runmplus access1-access8 country, ///

coverage(0) varnocheck ///

///cat() ///

variable( ///

classes=d(5); ///

knownclass=d(country); ///

usevariable = access1 access3-access8; ///

) analysis( ///

type=mixture; ///

estimator=bayes; ///

!fbiter=1000; ///

thin=40; ///

!chains=1; ///

alignment = free ; /// this makes the lv to N(0,1) in a reference dataset.

algorithm=integration; ///

) model( ///

%OVERALL% ///

RA1 by access1 access3-access8 ; ///

) output( align; ) ///stdyx;modindices;

savedata(save=fscores(10); file=fscores_dep1_pv.txt)
